# Correction to “Cortical Actin Depolymerisation in 3D Cell Culture Enhances Extracellular Vesicle Secretion and Therapeutic Effects”

**DOI:** 10.1002/jev2.70136

**Published:** 2025-08-06

Yang, Z., Li, X., Lin, Q., Zhou, F., Liang, K., Li, J.J., Niu, Y., Meng, Q., Zhao, T., Li, H., Wang, D., Lin, J., Li, H., Wang, B., Liu, W., Du, Y., Lin, J. and Xing, D. (2025), Cortical Actin Depolymerisation in 3D Cell Culture Enhances Extracellular Vesicle Secretion and Therapeutic Effects. J Extracell Vesicles., 14: e70109. https://doi.org/10.1002/jev2.70109

In the originally published article, the funding information was incorrect. The correct funding information is included below and has been updated in the online version of the article.

Incorrect

Funding: This work was funded by grants from Beijing Natural Science Foundation (L222087), Peking University Clinical Scientist Training Program (BMU2024PYJH015, supported by “the Fundamental Research Funds for the Central Universities”), Natural Science Foundation of China (82272538), and Innovation Fund for Outstanding Doctoral Students of Peking University Health Science Center.

Correct

Funding: This work was funded by the National Key Research and Development Program of China (Grant Number: 2024YFA1108603), Peking University Clinical Scientist Training Program, supported by the Fundamental Research Funds for the Central Universities (Grant Number: BMU2024PYJH015), National Natural Science Foundation of China (Grant Numbers: 82472482, 82272538), Innovation Fund for Outstanding Doctoral Students of Peking University Health Science Center, and the Natural Science Foundation of Beijing Municipality (Grant Number: L222087).

We apologize for this error.

